# Cocultivation of Chinese prescription and intestine microbiota: SJZD alleviated the major symptoms of IBS-D subjects by tuning neurotransmitter metabolism

**DOI:** 10.3389/fendo.2022.1053103

**Published:** 2022-11-14

**Authors:** Xiuwen Xia, Ya Xie, Qiaoqiao Chen, Dou Ding, Zongqin Wang, Yaji Xu, Yili Wang, Xiumin Wang, Weijun Ding

**Affiliations:** ^1^ School of Basic Medical Sciences, Chengdu University of Traditional Chinese Medicine, Chengdu, China; ^2^ Department of Fundamental Medicine, Neijiang Health Vocational College, Neijiang, China; ^3^ Department of Traditional Chinese Medicine, Zunyi Medical and Pharmaceutical College, Zunyi, China; ^4^ Department of Gastroenterology, Sichuan Hospital of Traditional Chinese Medicine, Chengdu, China; ^5^ Medical School, Chengdu University, Chengdu, China; ^6^ Innovative Institute of Chinese Medicine and Pharmacy, Chengdu University of Traditional Chinese Medicine, Chengdu, China; ^7^ Department of Proctology, Chengdu First People’s Hospital, Chengdu, China

**Keywords:** diarrhea-predominant irritable bowel syndrome (IBS-D), Si-Jun-Zi decoction (SJZD), nontargeted metabolomics, 16S rRNA gene sequencing, microbiota-herbal cocultivation

## Abstract

**Objective:**

Diarrhea-predominant irritable bowel syndrome (IBS-D) is a recurrent and common disease featuring dysbiotic intestinal microbiota, with limited treatments. Si-Jun-Zi Decoction (SJZD), a classic Chinese prescription, has been extensively used for IBS-D. This work aimed to explore the *ex vivo* interactions of SJZD and IBS-D’s intestinal microbiota.

**Methods:**

Five samples of intestinal microbiota collected from IBS-D volunteers and five age-matched healthy controls were recruited from the Affiliated Hospital, Chengdu University of Traditional Chinese Medicine (TCM). A representative mixture of intestinal microbiota was composed of an equal proportion of these fecal samples. To simulate the clinical interaction, this microbiota was cocultivated with SJZD at clinical dosage in an anaerobic incubator at 37°C for 35 h. Microbiota and metabolic alterations were assessed by 16S rRNA gene sequencing in the V3/V4 regions and a nontargeted metabolome platform, respectively.

**Results:**

After being cocultivated with SJZD, the dysbiotic intestine microbiota from IBS-D subjects was largely restored to those of the healthy controls. A total of 624 differentially expressed metabolites were detected by nontargeted metabolomics, of which 16 biomarkers were identified. These metabolites were then enriched into 11 pathways by KEGG, particularly those involved in neurotransmitter metabolism responses for the major symptom of IBS-D. Correlation analysis of bacterial metabolites demonstrated a synergistic pattern of neurotransmitter metabolism between *Streptococcus* and *E. Shigella.*

**Conclusion:**

*SJZD* rescued the dysbiotic intestinal microbiota and ameliorated the dysfunctional neurotransmitter metabolism involved in IBS-D’s major symptoms.

## Introduction

Irritable bowel syndrome (IBS) is a common and recurrent disease, with an internationally pooled prevalence of 12.41% and limited treatments (1, 2). Diarrhea-predominant irritable bowel syndrome (IBS-D), as the major type of IBS, is characterized by perennial abdominal pain and diarrhea (3), with complex and diverse pathogenesis (4–6). Although its etiology remains unclear, microbial factors play key roles in IBS pathophysiology (7). Both the structure and function of the intestinal microbiota of IBS-D patients are intensively disturbed (8, 9). The multifactorial pathophysiology of IBS-D suggests multiple therapeutic approaches, such as altering intestine microbiota, visceral hypersensitivity, intestinal permeability, gut-brain interaction and psychological strategies (10). Therefore, complementary and alternative medicines such as traditional Chinese medicine (TCM) featuring synergistic effects may show special activities for IBS-D (11).

Few investigations have shown direct interactions between TCM formulas and gut microbiota. It is well known that oral administration is the major routine of TCM that inevitably interacts with the intestinal microbiota. The slight alterations of the construction and function in gut microbiota can significantly change the decomposition, transformation, and absorption of complex ingredients in Chinese herbs. Hence, directly detecting the potential molecular interactions between the intestinal microbiota and TCM herbs is pivotal for probing their interactive mechanisms. For instance, Si-Jun-Zi Decoction (SJZD) has been extensively used for IBS-D and other intestinal complaints for hundreds of years (11, 12). Unfortunately, the underlying mechanisms of SJZD *via* intestinal microbiota against IBS-D have not been fully described.

The *ex vivo* fermentation system is a promising platform for exploring the direct interactions between TCM formulas and intestinal microbiota. Accumulating publications have demonstrated that gut microbiota can transform ingredients in TCM herbs into diverse metabolites that show different bioavailability, bioactivity and/or toxicity (13), which can intensively impact the health and disease of mammalian hosts (14). A considerable amount of research has been performed to explore the interactions between intestinal microbiota (always from healthy volunteers) and the ingredients of certain TCM herbs (15, 16). However, few studies have explored the precise interactions of intestinal microbiota derived from particular patients and relevant TCM formulas (17, 18). Originally from long-term successful clinical practice, TCM formulas consisting of several herbs create a much more complicated mechanism because different active ingredients can work together to produce a more desired health effect or can cancel out the negative effects associated with a single herb, thereby minimizing side effects and retaining only the desired effect.

In the present work, an *ex vivo* cocultivation system will be established to determine the direct interactions of SJZD with the intestinal microbiota derived from IBS-D patients (19). To simulate the interactive regulation process between IBS-D intestine flora and effective TCM formulas, this anaerobic fermentation platform is of scientific significance for elucidating the interactive molecular mechanisms of a large number of TCM prescriptions and sparks a revolution in drug discovery based on Chinese formulas (20).

## Materials and methods

### Collection and preparation of samples

This work was approved by the Ethics Committee of the Chengdu University of TCM. Each participant signed an informed consent form before the experiment. Five representative IBS-D patients (group IBS-D) were diagnosed and recruited according to TCM standards (ZY/T001.9~001.9.94 & GB/T16751.2~1997) for the screen of Spleen-deficiency syndrome and Rome III diagnostic criteria for IBS-D identification. Five age-matched healthy subjects were simultaneously enrolled as normal controls (NC group). All participants had a routine diet before sample collection. None of the volunteers had taken antibiotics for the last three months. All fecal samples were collected under sterile rule. A solution for sample preservation and transportation, composed of KH_2_PO_4_ (0.45%), Na_2_HPO_4_ (0.6%), Tween-80 (0.05%), and agar (0.1%), was prepared and applied for temporary storage and transit of collected samples.

### Preparation of SJZD solution

All herbs of SJZD formula were purchased from the Affiliated Hospital, Chengdu University of TCM. It consists of six herbs: *Codonopsis Tangshan Oliv.*, *Atractylodes macrocephala Koidz.*, *Poria cocos (Schw. Wolf*, *Glycyrrhiza uralensis Fisch*, *Citrus reticulata Blanco*, *Zingiber oj-jicinale Rosc.*, 30 g for each herb. This formula was prepared under the manufacturing rule of TCM decoction (21). The applied solution of SJZD was adjusted to 1 g/ml (W/V).

### Coculturation of SJZD with intestine microbiota

#### Reagents

Tween-80 (Kemiou Chemical reagent, Tianjin, China), vitamin K1 (Solarbio Biotechnology, Beijing, China), and protohemin (Meilun Biotech, Dalian, China) were used in anaerobic culture. The culture medium, named General Anaerobic Medium (GAM) (Hopebiol Biotechnology, Qingdao, China), was composed of peptone 5.0 g, proteose peptone 5.0 g, enzymatically digested soybean meal 3.0 g, serum powder 10.0 g, beef extract 2.2 g, yeast extract 2.5 g, liver infusion 1.2 g, soluble starch 5.0 g, dextrose 0.5 g, sodium chloride 3.0 g, monopotassium phosphate 2.5 g, L-tryptophan 0.2 g, L-arginine 1.0 g, sodium thioglycollate 0.3 g, and L-cysteine monohydrochloride 0.3 g, and water was added up to 1000 mL. pH 7.3 was adjusted, sterilized at 121 °C for 20 min, and stored at 4°C for culture. The culture solution (KH_2_PO_4_ 4.5 g, Na_2_HPO_4_ 6 g, Tween-80 0.5 g, agar 1 g, added distilled water to 1000 ml) was mixed with GAM 3:7 (V/V) to obtain the transfer solution.

#### Preparation of intestinal microbiota

Approximately 1 g of fecal sample was collected from each volunteer. The fecal sample was immediately put into a tube with 2 ml of the solution for sample preservation and transportation as described above. After slight mixing at 4°C for 5 min, 0.2 ml of the mixture was removed from each sample. The representative intestinal microbiota was then obtained by putting together five mixtures of groups IBS-D or NC and stored at -80°C.

#### Cocultivation

The representative intestinal microbiota derived from the IBS-D and NC groups were resuscitated in GAM (1:9, V/V) at 37°C. A total of 10 ml of gut microbiota was mixed with 90 ml of drug-containing GAM (1:4, V/V) and cultured in a 250 ml flask located in an anaerobic airbag at 37°C for 35 h. NC0, NC6, NC12, NC24 and NC35 represent the coculture mixtures from group NC and sampled at 0, 6, 12, 24, and 35 h postincubation, respectively, while IBSD0, IBSD6, IBSD12, IBSD24, and IBSD35 represent those of group IBS-D. Three duplicated samples of each group were performed and centrifuged at 4000 g and 4°C for 10 min. The supernatant was collected, and three samples at each time point were equally mixed together and stored in a -80°C refrigerator.

#### 16S rRNA gene sequencing

Cetyltrimethylammonium bromide (CTAB) and sodium dodecyl sulfate (SDS) were used to extract the total DNA from each sample of fermentation solution. The V3 and V4 regions of the 16S rRNA gene were amplified with a barcode (22) by a high-fidelity PCR master mix (New England Biolabs). The PCR products were then purified with a GeneJET gel extraction kit (Thermo Scientific). Sequencing libraries were generated using the NEB Next^®^ Ultra™ DNA Library Prep Kit for Illumina (NEB, USA) following the manufacturer’s recommendations, and index codes were added. Sequencing data were analyzed by a quantitative kit (Kapa Biosystems, KK4824) using an Illumina MiSeq platform for paired-end sequencing. The operational taxonomic units (OTUs) were selected through open reference OTU selection. Using LEfSe software, the LDA score was set to 4, and the community structure differences of the samples were analyzed. Metastatic analysis was carried out by R (Version 2.15.3) to analyze the differences between groups at each classification level, and the p value was obtained. Referring to Benjamin and Hochberg’s false discovery rate, the Q value was obtained by correction of the p value (23). R (Version 2.15.3) was used to perform the t test between groups to determine the species with significant differences between groups and draw the map.

#### Nontargeted metabolomics

Then, 400 μL of 80% methanol solution was added to 100 μL of fermentation, vortexed well, and centrifuged at 14000 g and 4°C for 20 min. Next, 300 μL of the supernatant was placed in a 1.5-ml centrifuge tube for LC–MS analysis. The supernatant was diluted to a final concentration of 53% methanol with LC–MS grade water. The samples were subsequently transferred to a fresh tube and centrifuged at 14000 g and 4°C for 20 min. Finally, the supernatant was injected into the LC–MS/MS system for further analysis. A Conquer UHPLC system (ThermoFisher) and a track rap Q extraction series mass spectrometer (ThermoFisher) were used in positive and negative modes. The original data generated by UHPLC–MS/MS were processed by peak pairing, peak selection, and quantification of each metabolite using compound finder 3.1 (CD3.1, Thermo Fisher). Principal component analysis (PCA) and partial least squares discrimination analysis (PLS-DA) were used to analyze the significant differences in metabolites between the IBS-D and NC groups. Hierarchical clustering (HCA) and metabolite correlation analysis were used to reveal the relationship between metabolites and samples. Finally, the differentially expressed metabolites (DEMs) were matched with relevant biological processes based on the KEGG database.

#### Network pharmacology forecast

Network pharmacology forecasting was performed by the “BATMAN-TCM” (Bioinformatics Analysis Tool for Molecular mechANism of Traditional Chinese Medicine) (24) to predict the compounds and corresponding targets in SJZD (set score = 20, *P* <= 0.05). Compared with the Therapeutic Target Database (TTD) and Online Mendelian Inheritance in Man (OMIM), targets and compounds related to IBS(-D) were obtained. The pathways that contact related targets were retrieved through the Kyoto Encyclopedia of Genes and Genomes (KEGG) database. Finally, the network diagram was drawn using Cytoscape 3.8.2 software.

#### Statistical analysis

SPSS Software (V16.0; SPSS Inc., Chicago, IL) was used for statistical analyses. All data are expressed as the mean ± standard deviation (SD). Data were analyzed with one-way ANOVA. Differences between the groups were evaluated using Student’s *t* test. Data were considered to be statistically significant with each value *P < 0.05, **P < 0.01. The correlation analysis was based on the cor() function in R (v3.1.3), and the Pearson correlation coefficient R (1 ≥ R ≥ - 1) between all metabolites was calculated to analyze the correlation among the metabolites. The cor.test () function (FDR < 0.05) was used to test the significance of the correlation analysis.

## Results

From May 15 to August 04, 2018, ten stool samples, including five IBS-D subjects (IBS-D group) and five healthy subjects as normal controls (NC group), were collected from patients aged 18~35 years in the outpatient clinic of the Department of Gastroenterology, Sichuan Provincial Hospital of TCM.

### Differential microbiota between IBS-D and healthy individuals

**Figure 1:**
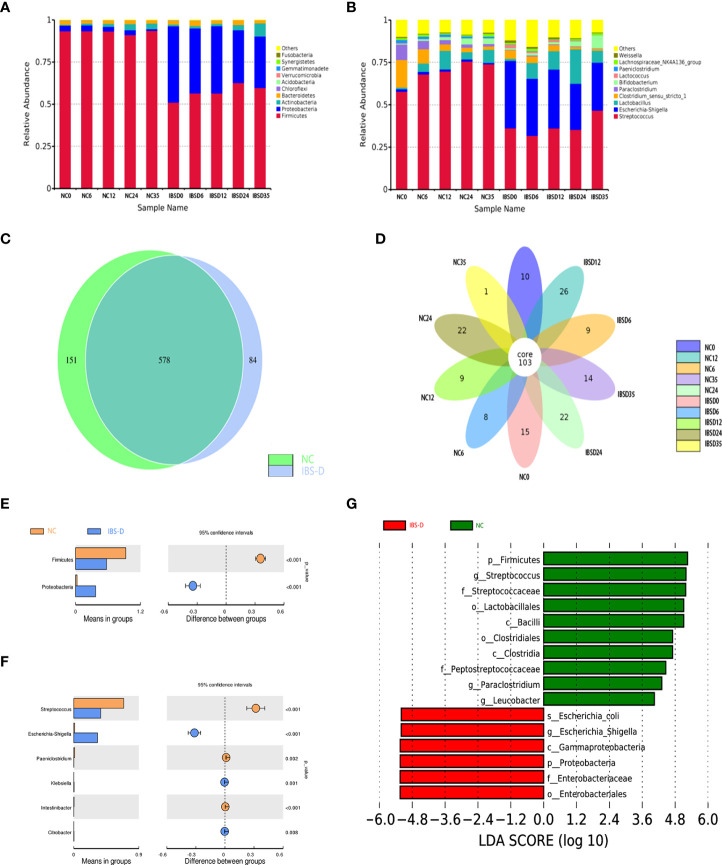
Basic characteristics of the cocultivated microbiota. **(A, B)** Histogram of relative abundance at the phylum **(A)** and genus **(B)** levels. The Arabic numerals within the sample name (0–35) indicate the culture time (hours). **(C, D)** Differentially expressed species between groups NC and IBS-D **(C)** and time series cocultivations **(D)**. **(E, F)** Differentially expressed taxa at the phylum **(E)** and genus **(F)** levels. **(G)** LEfSe analysis. LEfSe: LDA effect size analysis. LDA score: Linear discriminant analysis (LDA) affects the influence of species with significant differences in data. IBS-D: IBS-D group. NC: Normal control group. NC0-NC35: time series cocultivations of SJZD and intestine microbiota derived from the NC group. IBSD0-IBSD35: time series cocultivations of SJZD and intestine microbiota derived from the IBS-D group. SJZD: Si-Jun-Zi Decoction. IBS-D: diarrhea-predominant irritable bowel syndrome.

The results of 16S rRNA gene sequencing showed that the dominant phyla in the cocultivations were Firmicutes, Proteobacteria, Actinobacteria and Bacteroidetes (**Figure 1A**), while *Streptococcus, E. Shigella, Lactobacillus, Clostridium, Paraclostridium and Bifidobacterium* were *the main* genera (**Figure 1B**). A total of 578 OTUs were identified from both groups (**Figure 1C**), and 103 were identified as core bacteria (**Figure 1D**). The T tests between groups showed that *Proteobacteria* and *Firmicutes* were significantly different at the phylum level (**Figure 1E**), and 6 microbiota were significantly different at the genus level (**Figure 1F**). LEfSe analysis of biomarkers (gate level to genus level) in each group revealed 10 biomarkers in the IBS-D group and 6 biomarkers in the NC group (**Figure 1G**).

The alpha diversity counted by the Shannon and Simpson indexes between the IBS-D and NC groups was significantly different (*P < 0.05, **P < 0.01), while the Ace and Chao1 indexes were not (*P* > 0.05) (**Table 1** and **Figures S1A–D**). In addition, the beta diversities between the two groups also reached significant differences (**Figure S1E**).

**Table 1:** Alpha diversity analysis of the cocultivated intestinal microbiota.

Algorithm name	Wilcox (*p* value)	T test (*p* value)
Shannon	0.01587*	0.007274**
Simpson	0.007937**	0.001264**
Chao1	0.4206	0.3621
Ace	0.5476	0.4141

* *p* value <0.05, ** *p* value <0.01.

### SJZD restored the dysbiotic microbiota of IBS-D subjects

**Figure 2:**
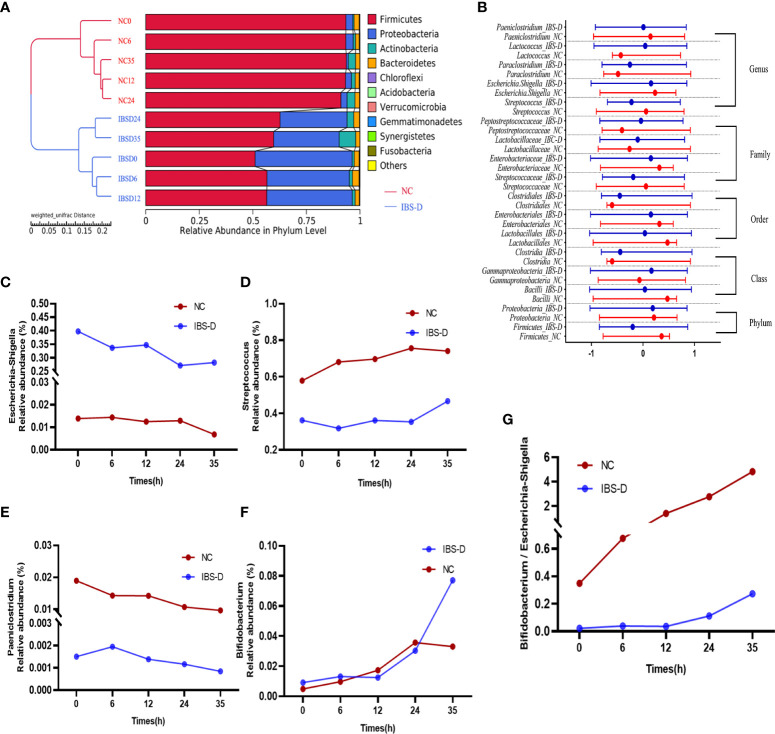
SJZD restored the dysbiotic intestinal microbiota of IBS-D subjects. **(A)** The unweighted pair-group method with arithmetic mean (UPGMA) clustering tree. **(B)** The Zscores of biomarkers at different taxa. The nodes in the graph show the median and interquartile ranges. Zscore = (x–μ)/σ (x: relative abundance of known bacteria, σ: standard difference, μ: average value). **(C-F)** Time series of relative abundances of *Escherichia-Shigella* **(C)**, *Streptococcus* **(D)**, *Paeniclostridium* **(E)**, and *Bifidobacterium* **(F)**. **(G)** *Bifidobacterium* to *Escherichia-Shigella ratio.* IBS-D: IBS-D group. NC: Normal controls. The Arabic numbers 0~35 indicate the coculture times (hours) of SJZD with the microbiota. SJZD: Si-Jun-Zi Decoction. IBS-D: diarrhea-predominant irritable bowel syndrome.

SJZD restores the gut microbiota of IBS-D *in vitro*. The UPGMA clustering tree shows that with the development of time, the clustering distance between IBS-D samples cultured for 35 h and the NC group becomes shorter, and the difference between the IBS-D group and the NC group decreases (**Figure 2A**). SJZD mainly reduced the relative abundance of the phylum *Proteobacteria* and increased the relative abundance of *Firmicutes* (**Figure 2A**). Based on the screened differential microbiota from T test and LEfSe analysis and observing their dynamics from 0 through 35 h coculture time. We homogenized the differential microbiota using the Zscore to assess changes in microbiota abundance at different taxonomic levels in the samples before and after culture and found that most microbiota in the IBS-D group were more discrete than those in the NC group (**Figure 2B**). Among the top 10 genera, *Escherichia-Shigella* (**Figure 2C**), *Streptococcus* (**Figure 2D**), and *Paeniclostridium* (**Figure 2E**) were upregulated by SJZD. In addition, SJZD-upregulated *Bifidobacterium* was also the dominant group in the Top10 at the genus level (**Figure 2F**). The change in the ratio of *Escherichia-Shigella* to *Bifidobacterium* in the IBS-D group was less than that in the NC group (**Figure 2G**).

### IBS-D-derived metabolites are involved in neuroactive ligand–receptor interactions

The potential targets of 450 compounds of SJZD were predicted by the BATMAN-TCM platform (**Table 2**). We then compared the TTD database with the OMIM database to select compounds and targets associated with IBS (**Table 3**). Third, five target genes (HTR3A, HTR4, HTR6, CRHR1, HTR3B) of SJZD acting on IBS-D were screened after comparing the above results. Finally, these target genes were enriched in four pathways (i.e., serotonergic synapse, calcium signaling pathway, neuroactive ligand–receptor interaction, long-term depression.) based on the KEGG database.

**Table 2:** Target prediction of IBS-D based on SJZD.

Gene name	Compound
HTR3A	1,22-Docosanediol; 1-Heptadecanol; Nicotine; Isotrilobine; Ergotamine; Phenylic Acid; Hesperidin; N-Nonanol; Isoliensinine; Tetrahydropalmatine
HTR4	Ergotamine; Hesperidin
HTR6	Nicotine; Ergotamine; Tetrahydropalmatine; Alpha-Curcumene
CRHR1	Ergotamine
HTR3B	1,22-Docosanediol; 1-Heptadecanol; Ergotamine; Phenylic Acid; N-Nonanol

**Table 3:** Targets to IBS predicted by the TTD and OMIM databases.

Term ID	Term description	gene names
hsa04726	Serotonergic synapse	HTR3A; HTR4; HTR6; HTR3B
hsa04020	Calcium signaling pathway	HTR4; HTR6
hsa04080	Neuroactive ligand–receptor interaction	HTR4; HTR6; CRHR1
hsa04730	Long-term depression	CRHR1

**Figure 3:**
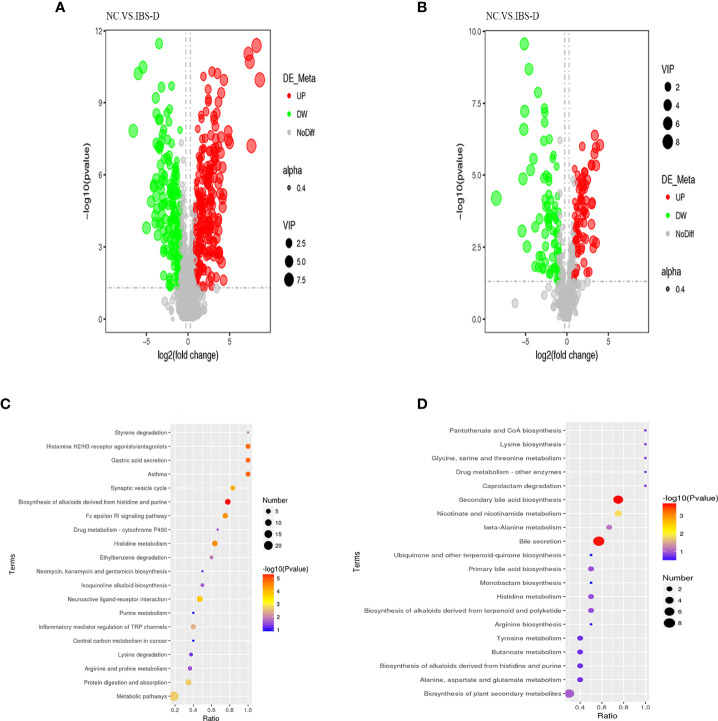
Nontargeted metabolites of the cocultured SJZD and intestine microbiota. **(A, B)** Volcano map of differentially expressed metabolites (DEM) from positive ion mode **(A)** and negative ion mode **(B)**. VIP: Variable importance in the projection. alpha: Transparency of dots. Black: the metabolite with no significant difference (nodiff), red: upregulated metabolite (up), green: downregulated metabolite (DW). **(C, D)** Bubble chart of enriched KEGG pathways, positive ion mode **(C)** and negative ion mode **(D)**. SJZD: Si-Jun-Zi Decoction. IBS-D: diarrhea-predominant irritable bowel syndrome.

Associated with the above virtual experiments, we analyzed the metabolites in the coculture system and found that the differentially expressed metabolites (DEM) were also enriched in neuroactive ligand–receptor interactions. A total of 458 DEMs (**Figure 3A, C**) were detected in positive ion mode, and 166 DEMs (**Figure 3B, D**) were detected in negative ion mode (**Table 4**). Eleven KEGG pathways were statistically enriched based on the above DEMs (p<0.05), including 14 metabolites such as histamine, morphine, cytocine, tryptamine, and pyridoxamine (**Table 5**). It is worth mentioning that histamine, morphine and tryptamine are enriched in the neuroactive ligand–receptor interaction pathway.

**Table 4:** Differentially expressed metabolites identified by LC–MS.

Compared samples	Num. of total ident.	Num. of total sig.	Num. of Sig.down	Num. of Sig.up
IBSD.vs.NC_pos	4042	458	262	196
IBSD.vs.NC_neg	1247	166	83	83

Num. Of Total Ident.: the total number of identified compounds. Num. Of Total Sig.: the number of metabolites detected as differentially expressed metabolites (DEM). Num. Of Sig.up: Number of upregulated DEMs. Num. Of Sig.down: the number of downregulated DEMs.

**Table 5:** KEGG pathways enriched by differentially expressed metabolites.

Map ID	Map title	Adjusted *p* value	Metabolite names
map01065	Biosynthesis of alkaloids derived from histidine and purine	0.000304047	Histamine; L-Histidine
map04971	Gastric acid secretion	0.000304047	Histamine
map05310	Asthma	0.000304047	Histamine
map07227	Histamine H2/H3 receptor agonists/antagonists	0.000304047	Histamine
map00340	Histidine metabolism	0.000479862	Histamine, L-Histidine
map04664	Fc epsilon RI signaling pathway	0.000479862	Histamine, Arachidonic acid
map04721	Synaptic vesicle cycle	0.000954972	Histamine
map04080	Neuroactive ligand–receptor interaction	0.001591021	Histamine, Morphine, Tryptamine
map04974	Protein digestion and absorption	0.015358943	Histamine, L-Histidine, Putrescine
map04750	Inflammatory mediator regulation of TRP channels	0.025307978	Histamine, Arachidonic acid
map01100	Metabolic pathways	0.015358943	Histamine, L-Histidine, Morphine, Tryptamine, Arachidonic acid, Putrescine, Guanine, Cytosine, Styrene, L-Tyrosine, Pipecolic acid, 1-Piperideine, Pyridoxamine, Agmatine

Mapid: the ID of the enriched KEGG pathway. Maptitle: The name of the enriched KEGG pathway. Adjusted pV: corrected p value. Meta names: enriched in different metabolites related to this pathway. KEGG pathway significant enrichment.

### Neurotransmitter-related metabolites in cocultivations involved in IBS-D therapy

**Figure 4:**
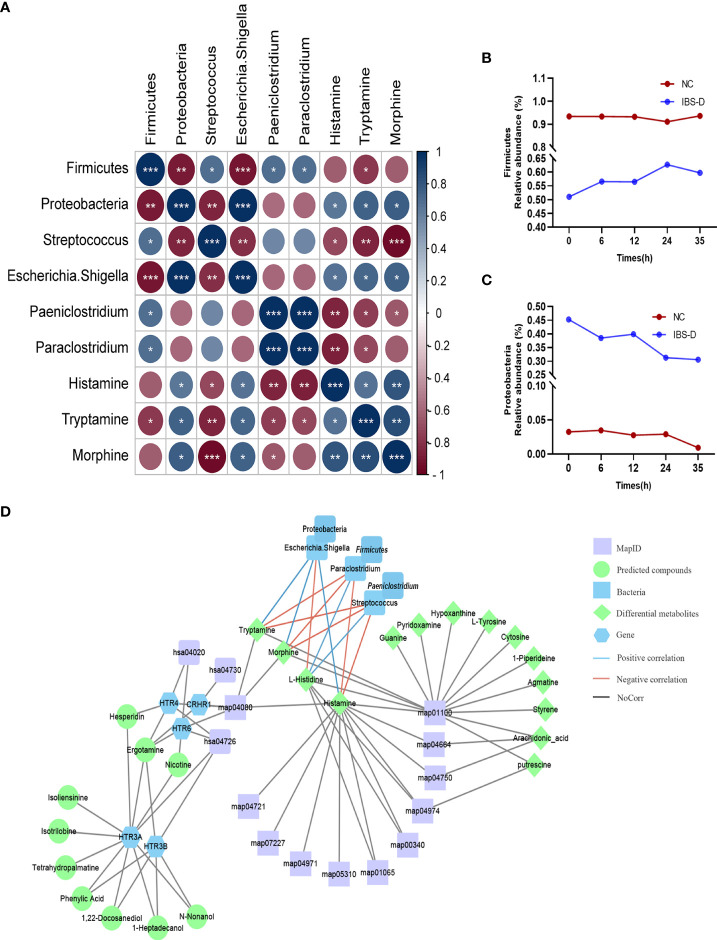
Significant correlations between microbiota and metabolites. **(A)** Correlation analysis of intestinal microbiota and differentially expressed metabolites. **(B, C)**. Time series of relative abundances of the phyla Proteobacteria **(B)** and Firmicutes **(C)** in a cocultivation system. **(D)** Compound-target network based on IBS-D microbiota-metabolome-SJZD compound analysis. SJZD: Si-Jun-Zi Decoction. IBS-D: diarrhea-predominant irritable bowel syndrome.

The gut microbiota showed a close correlation with intestinal DEMs (S2). Correlation analysis showed that five genera, *Proteobacteria* and *Paeniclostridium*, were significantly correlated with three DEMs (histamine, morphine and tryptamine) (**Figure 4A**). At the phylum level, SJZD mainly affected the relative abundance of *Firmicutes* (**Figure 4B**) and *Proteobacteria* (**Figure 4C**) in IBS-D subjects; *Proteobacteria* was negatively correlated with *Firmicutes* and positively correlated with histamine, morphine and tryptamine. At the genus level, *Escherichia-Shigella* was negatively correlated with *Streptococcus* and *Paeniclostridium* and positively correlated with histamine, morphine and tryptamine. Using Cytoscape 3.8.2 software, the crucial intestinal microbe-metabolite-SJZD-target network diagram was drawn (**Figure 4D**), particularly associated with the neuroactive ligand–receptor interaction pathway (hsa04080) and key intestinal microbiota.

## Discussion

Given that oral administration is the main approach in TCM, observation of the direct interactions between TCM formulas and gut microbiota is an extremely important and urgent research field. The intestinal microbiome of human beings is a diverse and dynamic collection of microorganisms that have much more metabolic potential than those of their mammalian host. Ever-increasing evidence indicates that the intestinal microbiota plays a pivotal role in TCM therapy by complicated interplay with Chinese components (25). This interplay includes activities such as intestinal microbiota biotransforming TCM components into metabolites with different bioavailability and bioactivity/toxicity from their precursors (15), improving the dysbiotic microbiota and consequently ameliorating associated pathological conditions (16) and mediating the synergistic and antagonistic interactions between the multiple chemicals in certain TCM formulas (26). However, the interactive mechanisms between TCM herbs/formula and relevant intestinal microbiota have always been distorted by gut ecology, such as mucosal immunity, the enteric nervous system and the hormonal environment.

This work aims to observe the direct interaction mechanism of a typical dysbiotic intestine microbiota and a TCM formula based on a cocultivation or fermentation system. IBS is a common but complex disease characterized by dysbiotic intestinal microbiota. Compared with healthy controls, the family *Enterobacteriaceae* (phylum *Proteobacteria*), family *Lactobacillaceae*, and genus *Bacteroides* were increased in IBS subjects, whereas *uncultured Clostridiales I*, genus *Faecalibacterium* and genus *Bifidobacterium* were decreased (27). On the other hand, the TCM formula SJZD has a long history of clinical application for functional dyspepsia and IBS-D. Therefore, we collected five intestinal microbiota samples derived from representative IBS-D subjects and five controls and established an *ex vivo* concultivation system to reveal the interaction pattern of SJZD compounds and the intestinal microbiota of IBS-D patients.

The results of 16S rRNA gene sequencing showed that SJZD effectively rescued intestinal dysbiosis in patients with IBS-D. Both alpha and beta diversities between the IBS-D and NC groups reached significant differences (**Table 1** and **Figure S1E**) (28–31). Core taxa were observed in both groups (**Figure 1B**), consistent with other publications (32). The genera *Bifidobacteria* (33), *Lactobacillus* and *Streptococcus* (34) can ameliorate the symptoms of IBS; we observed that the relative abundances of these genera increased with cocultivation time. Interestingly, the abundance of the genus *Streptococcus* was significantly higher than that in the NC group at every time point. Previous studies have reported that the abundance of Streptococcus in constipated IBS (IBS-C) is relatively high (35), but the underlying mechanism is not clear. IBS-D is associated with increased abundances of *Escherichia-Shigella (* 36–38). *Paeniclostridium* is related to intestinal injury and inflammation (39), whereas *Proteobacteria* is a negative factor for intestinal homeostasis (30–32). In summary, SJZD effectively rescued key abnormal bacteria in the intestinal microbiota of IBS-D patients.

Metabolome analysis revealed that SJZD beneficially tuned the altered metabolite profile of intestinal microbiota in IBS-D subjects. Abdominal pain, as one of the predominant manifestations of IBS-D, is associated with the abnormal metabolism of enteric neurotransmitters such as tryptophan. Tryptamine, as the bacterial metabolite of tryptophan, plays a pivotal role in balancing intestinal immune tolerance and maintaining intestinal microbiota (40–44) and promoting intestinal functions (45). In this work, we observed significantly higher levels of tryptamine, morphine, arachidonic acid, and histamine in the IBS-D group (S3). The concentrations of these neurotransmitters and metabolites were positively correlated with *Escherichia-Shigella* but negatively correlated with *Streptococcus* (**Figures 4B, D**). Although morphine has the effect of slowing down movement in the large intestine (46) and arachidonic acid may improve gastrointestinal movement (47), our present work showed that the content of morphine is not statistically high in the IBS-D group. The histamine level was negatively correlated with L-histidine (**Figures 4B, C**), suggesting that histidine was converted to histamine in the IBS-D group (S3). In addition, our results indicated that the genera *Escherichia-Shigella* and *Streptococcus* may synergistically regulate histidine metabolism and suggest that foods rich in L-histidine may worsen the symptoms of IBS-D. In a sentence, histamine, morphine and tryptamine, as crucial metabolites enriched in the neuroactive ligand–receptor interaction pathway, were the essential neurotransmitters derived from the fermentation of SJZD and the intestinal microbiota of IBS-D subjects. These metabolites respond to abdominal pain, the crucial symptom of IBS-D patients. Therefore, our results indicated that SJZD facilitated the *ex vivo* modulation of the metabolite profiles, particularly those involved in the pathway of neuroactive ligand–receptor interaction.

Correlation analysis further revealed the network outline of intestine microbiota, intestine metabolites, SJZD compounds and targets for IBS-D patients (**Figure 4D**), particularly associated with the neuroactive ligand–receptor interaction pathway (hsa04080) and core taxa in the intestinal microbiota. The BATMAN-TCM network pharmacology and other correlation analysis platforms revealed that, in addition to metabolic modulation of the intestinal microbiota that leads to symptom amelioration, SJZD compounds showed regulatory targets for IBS-D therapy. Specifically, the correlation analysis demonstrated that five genera were significantly correlated with IBS-D-relevant neurotransmitters (i.e., histamine, morphine and tryptamine) (**Figure 4A**). These results could be used to determine the correlation between *SJZD* and the restoration of abnormal abundances of certain intestinal microbes. This finding indicates that the cocultivation of *SJZD* and gut microbiota originating from IBS-D volunteers is a useful platform to explore the direct interactions of TCM formula and complex intestine microbiota.

Some shortcomings exist in this work. First, our concultivation platform does not fully mimic the intestine microecology of human beings. For instance, apart from the majority of anaerobic bacteria, there are facultative and even aerobic bacteria living within our intestine. The dynamic growth of anaerobic, facultative and aerobic bacteria is one of the key mechanisms underlying the robust intestinal air environment. Hence, our anaerobic culture environment cannot fully simulate clinical gas conditions. Second, although we used an ex vivo fermentation system and observed a direct interaction between SJZD and the intestinal microbiota of IBS-D patients, the underlying molecular regulation mechanisms need to be further explored. Third, to detect more complete mechanisms of SJZD against IBS-D subjects, comparative studies between *in vivo* and *ex vivo* models should also be executed. Finally, the representative samples of intestine microbiota were composed of only ten donors in this study. Given the marked individual diversities of the intestinal microbiota in IBS-D patients, a larger sample size might be required for further studies.

## Conclusion

Our work demonstrated that *SJZD* rescued the dysbiotic intestinal microbiota and ameliorated the dysfunctional neurotransmitter metabolism involved in the major symptoms of IBS-D. The *ex vivo* coculture system could be extensively used to reveal the direct interactions between TCM formulas and complex gut microbiota.

## Data availability statement

The original contributions presented in the study are publicly available. This data can be found here: NCBI database; PRJNA898776; https://www.ncbi.nlm.nih.gov/bioproject/PRJNA898776.

## Ethics statement

The studies involving human participants were reviewed and approved by The Ethics Committee of the Chengdu University of TCM. The patients/participants provided their written informed consent to participate in this study.

## Author contributions

XWX: Writing - Original Draft, Data Curation. YX: Investigation, Data Curation, Visualization. QQC: Visualization. DD: Writing-Review and Editing. ZQW: Investigation. YJX: Investigation. YLW: Resources. XMW: Resources. WJD: Writing - Review and Editing, Project administration, Funding acquisition. All authors contributed to the article and approved the submitted version.

## Acknowledgments

The author would like to express their sincere gratitude to the experimental technical assistance provided by the Institute of Chinese Medicine Innovation, Chengdu University of Traditional Chinese Medicine.

## Conflict of interest

The authors declare that the research was conducted in the absence of any commercial or financial relationships that could be construed as a potential conflict of interest.

## Publisher’s note

All claims expressed in this article are solely those of the authors and do not necessarily represent those of their affiliated organizations, or those of the publisher, the editors and the reviewers. Any product that may be evaluated in this article, or claim that may be made by its manufacturer, is not guaranteed or endorsed by the publisher.
