# Supplementary material for: Cocultivation of Chinese prescription and intestine microbiota: SJZD alleviated the major symptoms of IBS-D subjects by tuning neurotransmitter metabolism
Source: Front Endocrinol (Lausanne). 2022 Nov 14;13:1053103. doi: 10.3389/fendo.2022.1053103 (PMC9704418; doi:10.3389/fendo.2022.1053103)
Supplement: Supplementary file 1 [file DataSheet_1.docx]

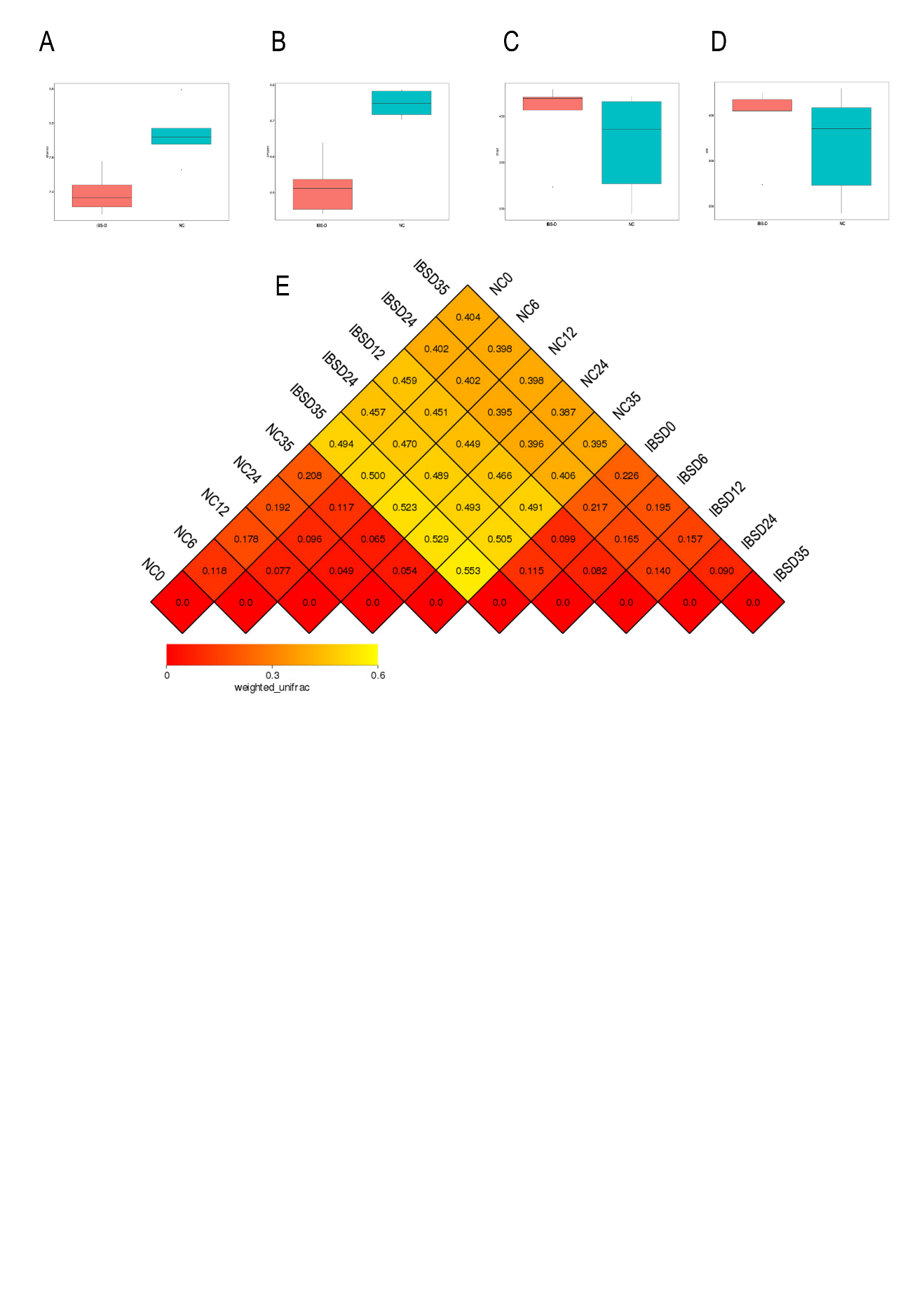


**Supplementary** **Figure 1.** Alpha diversity and beta diversity. **(A)** Shannon, **(B)** Simpson, **(C)** Chao1, **(D)** Ace, and **(E)** Sample Distance Heatmap. The beta diversity distance algorithm was used to obtain the distance matrix between samples and plot the distance heatmap between samples by R software.


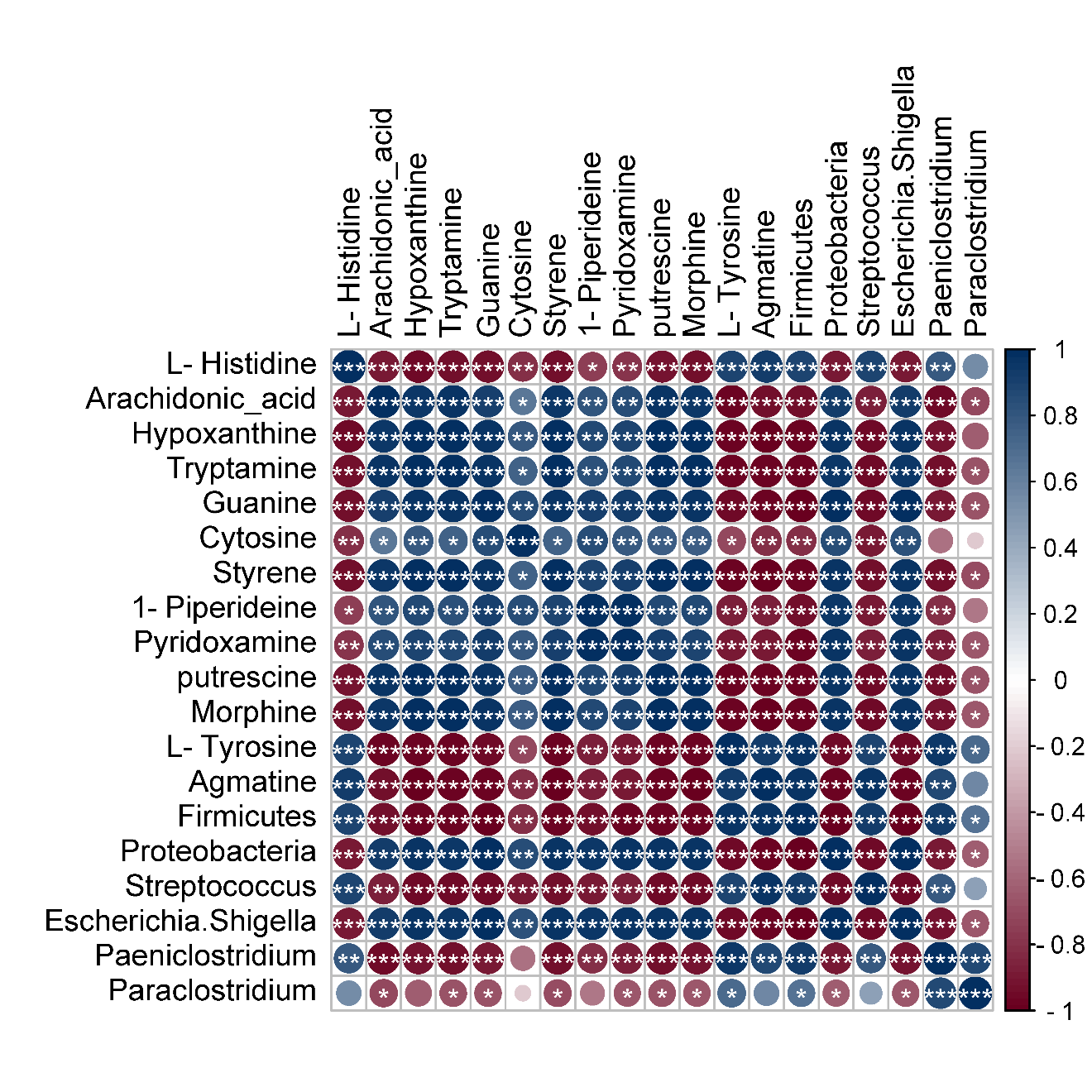


**Supplementary Figure 2.** Correlation analysis of 14 differentially expressed metabolites and the top 10 microbiota in relative abundance at the genus level. Red: negative correlation; Blue: positive correlation. * *P* < 0.05, ** *P* < 0.01, *** *P* < 0.001.

**
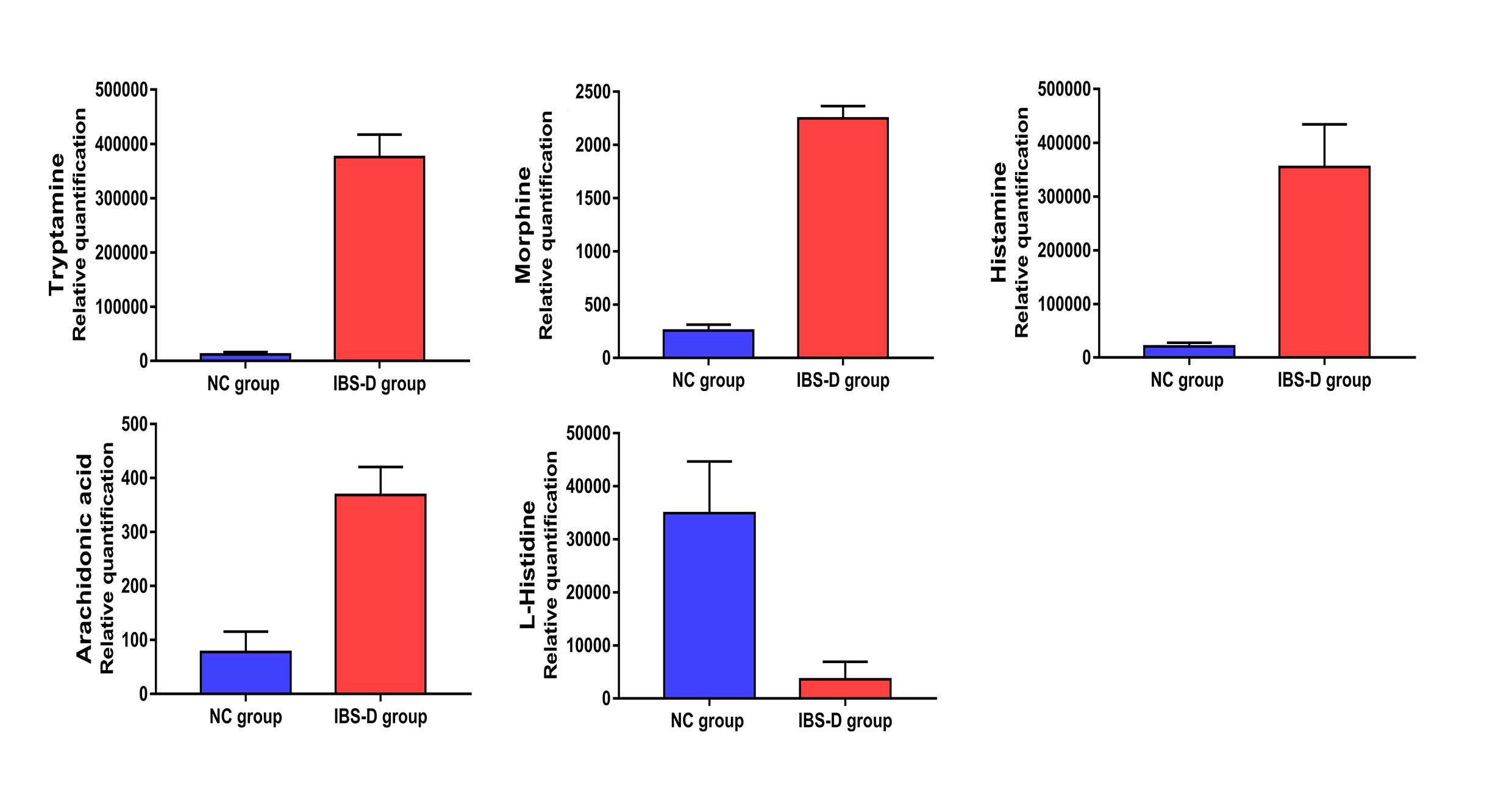
**

**Supplementary Figure 3.** Histogram of relative quantification of metabolites (*P* value < 0.05).
